# Is secondhand smoke associated with stress in smokers and non-smokers?

**DOI:** 10.1186/s12889-015-2612-6

**Published:** 2015-12-17

**Authors:** Seung Ju Kim, Kyu-Tae Han, Seo Yoon Lee, Sung-Youn Chun, Eun-Cheol Park

**Affiliations:** Department of Public Health, Graduate School, Yonsei University, Seoul, Republic of Korea; Institute of Health Services Research, Yonsei University, Seoul, Republic of Korea; Department of Health Policy and Management, Graduate School of Public Health, Yonsei University, Seoul, Republic of Korea; Department of Preventive Medicine, Yonsei University College of Medicine, 50 Yonsei-ro, Seodaemun-gu, Seoul, 120-752 Republic of Korea

**Keywords:** Secondhand smoke, Public health, Stress, Mental health

## Abstract

**Background:**

Secondhand Smoking (SHS) has been suggested as a major health problem in the world and is known to cause various negative health effects that have in turn caused the deaths of almost 600,000 people per year. Evidence has suggested that SHS may have an effect on health problems and such findings have influenced the implementation of smoking-free areas. However, few studies have investigated the effects of SHS on stress which is considered major risk factor for mental health. Thus, the purpose of our study was to investigate the association between exposure to SHS and stress.

**Methods:**

We performed a cross-sectional study using data from the Korea National Health and Nutrition Examination Survey (2007–2012). In our study, a total of 33,728 participants were included to evaluate the association between SHS exposure and stress based on smoking status. Association between SHS exposure and stress was examined using logistic regression models.

**Results:**

A total of 12,441 participants (42.9 %) were exposed to SHS in the workplace or at home. In our study, exposure to SHS was significantly associated with higher stress compared to non-exposure, regardless of smoking status (smoker odds ratio [OR]: 1.22; ex-smoker OR: 1.25; never-smoker OR: 1.42). Our results showed that the effect of SHS on stress was greater when exposure took place both at home and in the workplace in smokers and never-smokers.

**Conclusions:**

Exposure to SHS in the workplace and at home is considered to be a risk factor for high stress in both smokers and never-smoker. Therefore, strict regulations banning smoke which can smoking ban reduce SHS exposure are recommended in order to improve the populations’ health.

## Background

Smoking is a major problem worldwide that affects the health of individuals and may lead to disease or death. Worldwide, more than 5 million of deaths per year are caused by direct smoking, and 600,000 people have also died from the effects of second-hand smoke (SHS) [1]. Furthermore, smoke-related mortality has increased throughout the 21^st^ century. If no regulations are implemented to ban smoking, smoke-related deaths will increase to more than 8 million per year by 2030 [2]. Therefore, many countries have implemented policies that ban smoking in public places to protect people from SHS exposure [3].

In Korea, the Health Promotion Act was enacted in 1995; this included smoking bans in public buildings and places [4, 5]. However, smoking restrictions in buildings were limited to those of certain sizes (1998: over 3000 m^2^ ➔ 2006: over 1000 m^2^), and there has been only slight progress in designating non-smoking areas [5]. In 2010, it was possible to designate non-smoking areas, and fines could be imposed by local government ordinances. Smoke-free areas expanded to public places, and warning labels have had to be added to tobacco packaging since 2012. However, these efforts to reduce smoking have not had a large effect on smoking rates and the SHS exposure rate in Korea. According to Statistics Korea, there has been only a slight difference in the smoking rate between 2007 (25.3 %) and 2013 (24.1 %; smokers: daily or intermittent smokers over 19 years old) [6]. In addition, nearly half of all nonsmokers (including ex-smokers and never-smokers) have been exposed to SHS in the workplace according to data from 2007 (45.9 %) and 2013 (47.3 %) [6].

Recently, new smoke-free policies have resulted in major reductions in both the smoking rate and SHS exposure. First, the price of tobacco was increased from 2500 KRW to 4500 KRW in 2015. Over the past decade, there had been no changes in the tobacco price in Korea. Second, non-smoking areas were expanded so as to include all restaurants. Reductions in the smoking rate and exposure to SHS are expected; however, there have been disputes regarding the designation of smoke-free areas among policymakers and smokers, specifically whether these restrictions are excessive and infringe on the right to smoke [7].

Previous studies have shown that exposure to SHS can cause severe cardiovascular and pulmonary diseases, such as lung cancer, coronary heart disease, and heart failure [8–11]. Low infant birth weight and asthma in children may also occur if pregnant woman are exposed to SHS [12, 13]. In addition, SHS was associated with poor mental health, including depression and insufficient sleep [14–17]. Less is known, however, about the effect of exposure to SHS on stress. Particularly, evidence relating to the effects of SHS on mental health is lacking in Korea.

Stress is caused by various factors, including external stressors, responses to the external environment, and physical reactions to certain circumstances [18, 19]. In general, stress is the result of physical reactions in each individual, and it is caused by changes in health [19–21]. Stress can raise negative effective states, such as anxiety and depression, which can influence physical disease or disease risk. Chronic exposure to stress is considered the most harmful, as it can result in long-term or permanent changes in the emotional, physiological, and behavioral responses that influence susceptibility to and the course of disease [22]. In addition, stress is suggested as a major risk factor for suicide; thus, it is particularly important in Korea, which has the highest suicide rate among Organization for Economic Cooperation and Development (OECD) countries [23–25].

Thus, the aim of our study was to provide evidence to policymakers that can be used to support the implementation of smoke-free areas by investigating the association between exposure to SHS and stress. Considering that reported stress differs according to smoking status, we analyzed smokers, ex-smokers, and never-smoker separately [26]. Furthermore, we investigated the effects of SHS on stress according to different exposure locations, such as in the workplace and at home.

## Methods

### Data and population

**Fig. 1:**
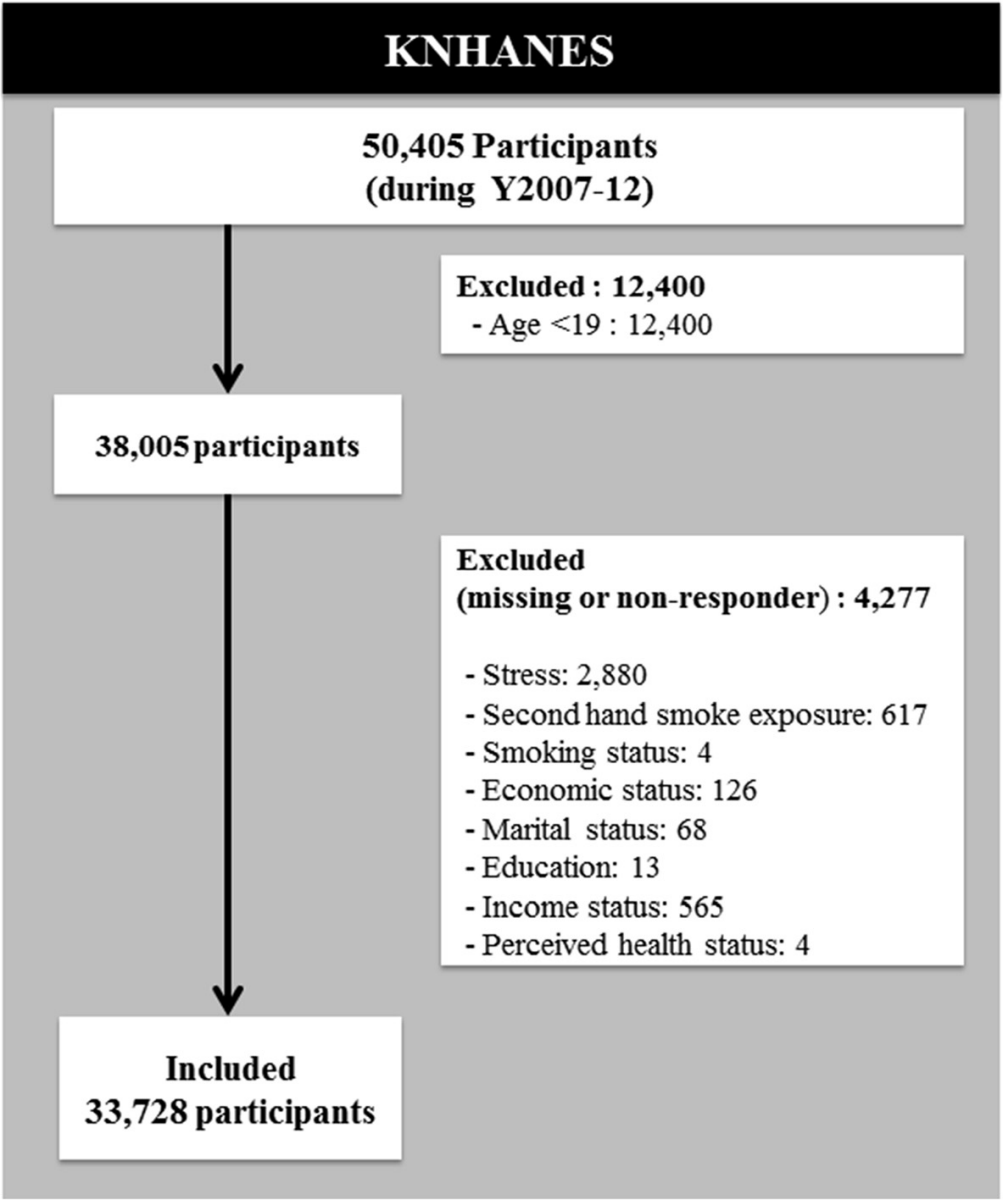
Flow diagram of the study participants

This cross-sectional study was performed using data from the 2007–2012 4^th^ and 5^th^ Korea National Health and Nutrition Examination Survey (KNHANES), which was performed by the Korea Center for Disease Control and Prevention. This nationwide cross-sectional survey has been conducted every year and has received approval from the Institutional Review Board at the Korea Center for Disease Control and Prevention since 2007 (2009-01CON-03-2C/2012-01-EXP-01-2C). The purpose of the KNHANES was to assess the health and nutritional status of Koreans and to provide data for evaluating health risk factors and other factors [27]. It was composed of a health interview, a health examination, and a nutrition survey, all of which were performed by trained medical staff and dieticians. Health interview questionnaires on individual factors such as smoking status, alcohol use, and mental health were collected via self-report. Other individual factors such as socioeconomic status, housing characteristics, and medical condition were collected via face-to-face interview. These data were collected from a total of 50,405 participants during the period of 2007–2012. From these data, we excluded subjects under 19 years old (*n* = 12,400) (i.e., those deemed too young to work), as we intended to assess exposure according to whether subjects were exposed in the workplace or at home. Next, we excluded missing values and non-responders from the data (*n* = 4277; see details in Fig. 1). A total of 33,728 participants were included in our study.

### Stress, smoking status and SHS

Stress was measured using self-reports that depended on typical stress recognition following the question: “Do you feel stress in your usual life activities?” Based on the self-report, stress was measured on a scale of 1 to 4 (1 = almost no stress in daily life, 2 = a little stress, 3 = much stress, 4 = very much stress). We classified subjects into two groups: a high stress group (score of 3 or 4) and a low stress group (score of 1 or 2).

We classified smoking status as never-smoker, ex-smoker, or smoker. We defined “never-smoker” as a person who had never smoked. Ex-smokers were defined as those who had smoked in the past yet currently did not. Smokers were defined as those who smoked every day or intermittently.

Exposure to SHS was measured only in the workplace or at home. In the workplace, exposure was self-reported and measured based on answers to the question “How long are you exposed to SHS in your workplace during a day?” Exposure to SHS at home was measured using answers to the question “How long are you exposed to SHS at home during a day?” A status of non-exposure was assigned to participants who answered that they were exposed to 0 h of SHS for each question. In addition, participants who were not employed were also classified as having a status of non-exposure in the workplace. Similarly, subjects were classified as having a non-exposure status at home if they answered “No” to the question “Do you live with a smoker, excluding yourself?” We then divided exposure to SHS into four groups: workplace & home, workplace, home, and non-exposure. Participants were classified as having exposure to SHS regardless of the duration, frequency, or location of any exposure they encountered.

### Covariates

We included sex, marital status, age, income, economic status, education level, perceived health status (good or bad), and year (2007–2012) as covariates. Age was stratified into six groups, beginning with 19 years of age. Income was classified as low, middle-low, middle-high, or high. Economic status was classified as employed or unemployed. Education level was classified as elementary school, middle school, high school, or university. Perceived health status was classified as good or bad, based on the question: “What do you think about your health status?”.

### Statistical analysis

All analyses were conducted using SAS version 9.2. We weighted the sampling results to convey an accurate representation of the whole nation. The weights were calculated by accounting for the complex survey design, survey non-response, and post-stratification [27]. The participants were thus assumed to represent the Korean population after weighting the data. In the fully adjusted model, all variables were entered simultaneously. Baseline demographic and clinical characteristics were compared using chi-squared tests, and *p* < 0.05 was considered statistically significant. We used logistic regression analysis (SAS procedure: PROC SURVEYLOGISTIC) [28] to calculate odds ratios (ORs) with 95 % confidence intervals in order to determine the association between exposure to SHS and stress. The main outcome measured the association between stress and exposure to SHS regardless of location, according to smoking status. The second outcome evaluated the association between different exposure locations and stress according smoking status.

## Results

In our study, a total of 33,728 participants were included to assess the association between exposure to SHS and stress. Among of them, 7237 (26.9 %) reported that they were current cigarette smokers, 6738 (19.9 %) reported themselves as ex-smokers, and 19,753 (53.2 %) reported themselves as never-smokers. Particularly, the workplace was found to be a major SHS exposure location (Table 1).

**Table 1:** General Characteristics of participants by smoking status

								(unit = n, %)
		Smoking status						*p*-value
		Smoker		Ex-smoker		Never-smoker		
Secondhand smoke exposure state								
Exposure	Workplace & home	422	(7.1)	132	(2.4)	978	(6.0)	<.0001
	Workplace	2,875	(42.5)	2,160	(38.4)	3,382	(19.9)	
	Home	424	(5.8)	232	(3.7)	1,836	(10.2)	
Non-exposure		3,516	(44.5)	4,214	(55.5)	13,557	(63.9)	
Sex								
Male		6,120	(86.8)	5,634	(83.4)	2,668	(18.5)	<.0001
Female		1,117	(13.2)	1,104	(16.6)	17,085	(81.5)	
Marital status								
Married		5,789	(72.2)	6,154	(85.7)	17,183	(79.6)	<.0001
Single		1,448	(27.8)	584	(14.3)	2,570	(20.4)	
Age								
19–29		1,113	(22.6)	524	(13.2)	2,557	(20.8)	<.0001
30–39		1,852	(27.8)	1,026	(18.6)	3,689	(19.6)	
40–49		1,542	(23.2)	1,135	(22.4)	3,694	(21.7)	
50–59		1,216	(15.5)	1,214	(19.9)	3,676	(17.5)	
60–69		858	(6.5)	1,399	(13.8)	3,290	(11.0)	
≥ 70		656	(4.4)	1,440	(12.0)	2,847	(9.5)	
Income								
Low		2,134	(30.2)	1,513	(22.3)	4,649	(24.8)	<.0001
Middle-low		1,904	(25.7)	1,700	(25.8)	4,860	(24.8)	
Middle-high		1,674	(23.5)	1,784	(26.3)	5,055	(25.3)	
High		1,525	(20.6)	1,741	(25.5)	5,189	(25.1)	
Economic status								
Employed		5,510	(78.5)	4,388	(70.8)	10,207	(54.3)	<.0001
Unemployed		1,727	(21.5)	2,350	(29.2)	9,546	(45.7)	
Educational level								
Elementary school		1,295	(12.2)	1,661	(17.8)	6,138	(23.1)	<.0001
Middle school		841	(10.6)	844	(11.1)	2,048	(9.7)	
High school		2,933	(45.7)	2,230	(37.8)	6,283	(37.0)	
University & college		2,168	(31.5)	2,003	(33.4)	5,284	(30.2)	
Perceived health status								
Good		5,824	(82.8)	5,343	(82.5)	15,152	(79.8)	<.0001
Bad		1,413	(17.2)	1,395	(17.5)	4,601	(20.2)	
Year								
2007		543	(8.0)	561	(9.1)	1,511	(8.0)	<.0001
2008		1,457	(18.4)	1,240	(18.0)	3,732	(17.4)	
2009		1,650	(18.7)	1,406	(18.1)	4,318	(19.0)	
2010		1,329	(19.0)	1,252	(18.9)	3,570	(18.6)	
2011		1,208	(18.4)	1,177	(17.8)	3,262	(17.8)	
2012		1,050	(17.5)	1,102	(18.1)	3,360	(19.1)	
Total		7,237	(26.9)	6,738	(19.9)	19,753	(53.2)	

% weighted percentage

Among all participants, 1532 (5.6 %) were exposed to SHS both in the workplace and at home, and 564 (37.2 %) of these participants reported that they felt high stress. A total of 8417 (29.7 %) participants were exposed to SHS in the workplace only, and among them, 2678 (31.2 %) reported that they felt high stress. A total of 2492 participants were exposed to SHS at home only, and among them, 867 reported high stress. In addition, the proportion of participants who felt high stress was higher among smokers (*n* = 2306; 32.8 %) than among ex-smokers (*n* = 1552; 24.5 %; Table 2).

**Table 2:** General characteristics of participants according to stress

								(unit = n, %)
		Stress				Total		*p*-value
		High		Low				
Secondhand smoke exposure state								
Exposure	Workplace & home	564	(37.2)	968	(62.8)	1,532	(5.6)	<.0001
	Workplace	2,678	(31.2)	5,739	(68.8)	8,417	(29.7)	
	Home	867	(34.8)	1,625	(65.2)	2,492	(7.7)	
Non-exposure		5,145	(25.3)	16,142	(74.7)	21,287	(57.1)	
Smoking status								
Smoker		2,306	(32.8)	4,931	(67.2)	7,237	(26.9)	<.0001
Ex-smoker		1,552	(24.5)	5,186	(75.5)	6,738	(19.9)	
Non-smoker		5,396	(27.8)	14,357	(72.2)	19,753	(53.2)	
Sex								
Male		3,501	(25.7)	10,921	(74.3)	14,422	(49.8)	<.0001
Female		5,753	(31.2)	13,553	(68.8)	19,306	(50.2)	
Marital status								
Married		7,720	(27.5)	21,406	(72.5)	29,126	(78.8)	<.0001
Single		1,534	(32.2)	3,068	(67.8)	4,602	(21.2)	
Age								
19–29		1,454	(33.5)	2,740	(66.5)	4,194	(19.8)	<.0001
30–39		2,136	(32.9)	4,431	(67.1)	6,567	(21.6)	
40–49		1,804	(28.1)	4,567	(71.9)	6,371	(22.2)	
50–59		1,533	(24.4)	4,573	(75.6)	6,106	(17.5)	
60–69		1,210	(21.7)	4,337	(78.3)	5,547	(10.4)	
≥ 70		1,117	(23.4)	3,826	(76.6)	4,943	(8.6)	
Income								
Low		2,516	(31.0)	5,780	(69.0)	8,296	(25.8)	<.0001
Middle-low		2,375	(28.8)	6,089	(71.2)	8,464	(25.2)	
Middle-high		2,229	(26.7)	6,284	(73.3)	8,513	(25.0)	
High		2,134	(27.3)	6,321	(72.7)	8,455	(24.0)	
Economic status								
Employed		5,818	(29.6)	14,287	(70.4)	20,105	(64.1)	<.0001
Unemployed		3,436	(26.4)	10,187	(73.6)	13,623	(35.9)	
Educational level								
Elementary school		2,439	(27.2)	6,655	(72.8)	9,094	(19.1)	<.0001
Middle school		847	(23.9)	2,886	(76.1)	3,733	(10.2)	
High school		3,054	(27.7)	8,392	(72.3)	11,446	(39.5)	
University & college		2,914	(31.7)	6,541	(68.3)	9,455	(31.2)	
Perceived health status								
Good		6,189	(24.9)	20,130	(75.1)	26,319	(81.1)	<.0001
Bad		3,065	(43.9)	4,344	(56.1)	7,409	(18.9)	
Year								
2007		684	(27.2)	1,931	(72.8)	2,615	(8.2)	<.0001
2008		1,803	(28.8)	4,626	(71.2)	6,429	(17.8)	
2009		2,195	(31.0)	5,179	(69.0)	7,374	(18.8)	
2010		1,674	(28.1)	4,477	(71.9)	6,151	(18.7)	
2011		1,502	(28.1)	4,145	(71.9)	5,647	(18.0)	
2012		1,396	(26.9)	4,116	(73.1)	5,512	(18.4)	
Total		9,254	(28.5)	24,474	(71.5)	33,728	(100.0)	

% weighted percentage

The main results of the association between SHS exposure and stress were calculated according to smoking status. Regardless of smoking status, SHS exposure was more associated with higher stress than non-exposure after fully adjusted model. However, the effect of SHS exposure on stress was greater in never-smokers (OR: 1.42; 95 % CI: 1.30–1.56) than in smokers (OR: 1.22; 95 % CI: 1.08–1.39) and, similarly, ex-smokers (OR: 1.25; 95 % CI: 1.07–1.46). Regarding economic status, employed never-smokers felt higher stress than those who were unemployed (OR: 1.23; 95 % CI: 1.12–1.34; Table 3).

**Table 3:** The association between SHS exposure and stress by smoking status

	Unadjusted									Adjusted								
	Smoker			Ex-smoker			Never smoker			Smoker			Ex-smoker			Never smoker		
Variable	OR	95 % CI		OR	95 % CI		OR	95 % CI		OR	95 % CI		OR	95 % CI		OR	95 % CI	
Secondhand smoke exposure state																		
Exposure^a^	1.31	1.16	1.47	1.42	1.23	1.63	1.44	1.33	1.57	1.22	1.08	1.39	1.25	1.07	1.46	1.42	1.30	1.56
Non-exposure	1.00	-	-	1.00	-	-	1.00	-	-	1.00	-	-	1.00	-	-	1.00	-	-
Sex																		
Male	0.49	0.42	0.00	0.44	0.37	0.52	0.64	0.57	0.73	0.51	0.43	0.61	0.50	0.41	0.61	0.59	0.52	0.67
Female	1.00	-	-	1.00	-	-	1.00	-	-	1.00	-	-	1.00	-	-	1.00	-	-
Marital status																		
Married	0.90	0.78	1.03	0.70	0.57	0.86	0.82	0.74	0.91	1.22	1.01	1.46	0.99	0.74	1.32	1.08	0.91	1.29
Single	1.00	-	-	1.00	-	-	1.00	-	-	1.00	-	-	1.00	-	-	1.00	-	-
Age																		
19–29	1.88	1.44	2.45	2.50	1.92	3.26	1.29	1.12	1.49	2.69	1.86	3.89	2.54	1.67	3.84	1.98	1.57	2.52
30–39	2.01	1.57	2.57	2.56	2.06	3.18	1.13	0.98	1.29	2.61	1.87	3.63	2.68	1.98	3.62	1.51	1.25	1.83
40–49	1.60	1.26	2.05	1.93	1.56	2.39	0.96	0.84	1.09	2.03	1.48	2.78	2.30	1.72	3.08	1.25	1.04	1.50
50–59	1.19	0.91	1.54	1.39	1.11	1.75	0.90	0.79	1.03	1.45	1.08	1.95	1.66	1.27	2.17	1.08	0.92	1.25
60–69	0.81	0.61	1.08	0.92	0.72	1.16	0.93	0.81	1.07	0.96	0.70	1.30	1.06	0.82	1.36	1.02	0.88	1.18
70≥	1.00	-	-	1.00	-	-	1.00	-	-	1.00	-	-	1.00	-	-	1.00	-	-
Income																		
Bottom	0.90	0.76	1.07	1.41	1.14	1.74	1.26	1.13	1.41	0.87	0.72	1.04	1.30	1.03	1.63	1.22	1.08	1.38
Middle-bottom	0.86	0.72	1.02	1.22	1.00	1.49	1.13	1.01	1.26	0.84	0.70	1.01	1.18	0.96	1.46	1.10	0.98	1.24
Middle-top	0.85	0.72	1.00	0.91	0.74	1.11	1.05	0.94	1.18	0.84	0.70	1.00	0.87	0.70	1.08	1.05	0.93	1.18
Top	1.00	-	-	1.00	-	-	1.00	-	-	1.00	-	-	1.00	-	-	1.00	-	-
Economic status																		
Working	1.01	0.88	1.17	1.10	0.95	1.28	1.21	1.12	1.30	1.01	0.85	1.21	1.20	0.99	1.45	1.23	1.12	1.34
Not working	1.00	-	-	1.00	-	-	1.00	-	-	1.00	-	-	1.00	-	-	1.00	-	-
Educational level																		
Elementary school	0.61	0.51	0.73	0.72	0.60	0.86	0.93	0.85	1.02	0.77	0.60	0.98	1.03	0.80	1.34	0.89	0.76	1.03
Middle school	0.72	0.58	0.89	0.63	0.49	0.81	0.66	0.58	0.76	0.82	0.64	1.04	0.83	0.63	1.11	0.67	0.57	0.79
High school	0.81	0.71	0.92	0.88	0.75	1.03	0.80	0.73	0.88	0.78	0.68	0.90	0.84	0.71	1.01	0.76	0.68	0.84
University & college	1.00	-	-	1.00	-	-	1.00	-	-	1.00	-	-	1.00	-	-	1.00	-	-
Perceived health status																		
Good	0.45	0.39	0.52	0.54	0.46	0.64	0.37	0.34	0.41	0.39	0.34	0.46	0.45	0.38	0.55	0.33	0.30	0.36
Bad	1.00	-	-	1.00	-	-	1.00	-	-	1.00	-	-	1.00	-	-	1.00	-	-
Year																		
2007	1.00	-	-	1.00	-	-	1.00	-	-	1.00	-	-	1.00	-	-	1.00	-	-
2008	1.18	0.92	1.51	1.41	1.06	1.87	0.93	0.79	1.09	1.14	0.89	1.46	1.39	1.02	1.88	0.87	0.73	1.02
2009	1.18	0.92	1.50	1.39	1.06	1.83	1.15	0.99	1.33	1.20	0.94	1.53	1.38	1.04	1.83	1.12	0.96	1.31
2010	1.07	0.82	1.40	1.27	0.95	1.70	0.95	0.81	1.12	1.10	0.84	1.45	1.25	0.92	1.71	0.92	0.77	1.09
2011	1.16	0.89	1.51	1.01	0.75	1.36	0.98	0.84	1.15	1.22	0.93	1.60	1.06	0.77	1.46	1.00	0.84	1.18
2012	1.02	0.79	1.34	1.19	0.88	1.59	0.90	0.77	1.05	1.02	0.78	1.34	1.26	0.92	1.73	0.92	0.78	1.09

^a^Exposure included participants who were exposed to SHS in the workplace or at home

**Fig. 2:**
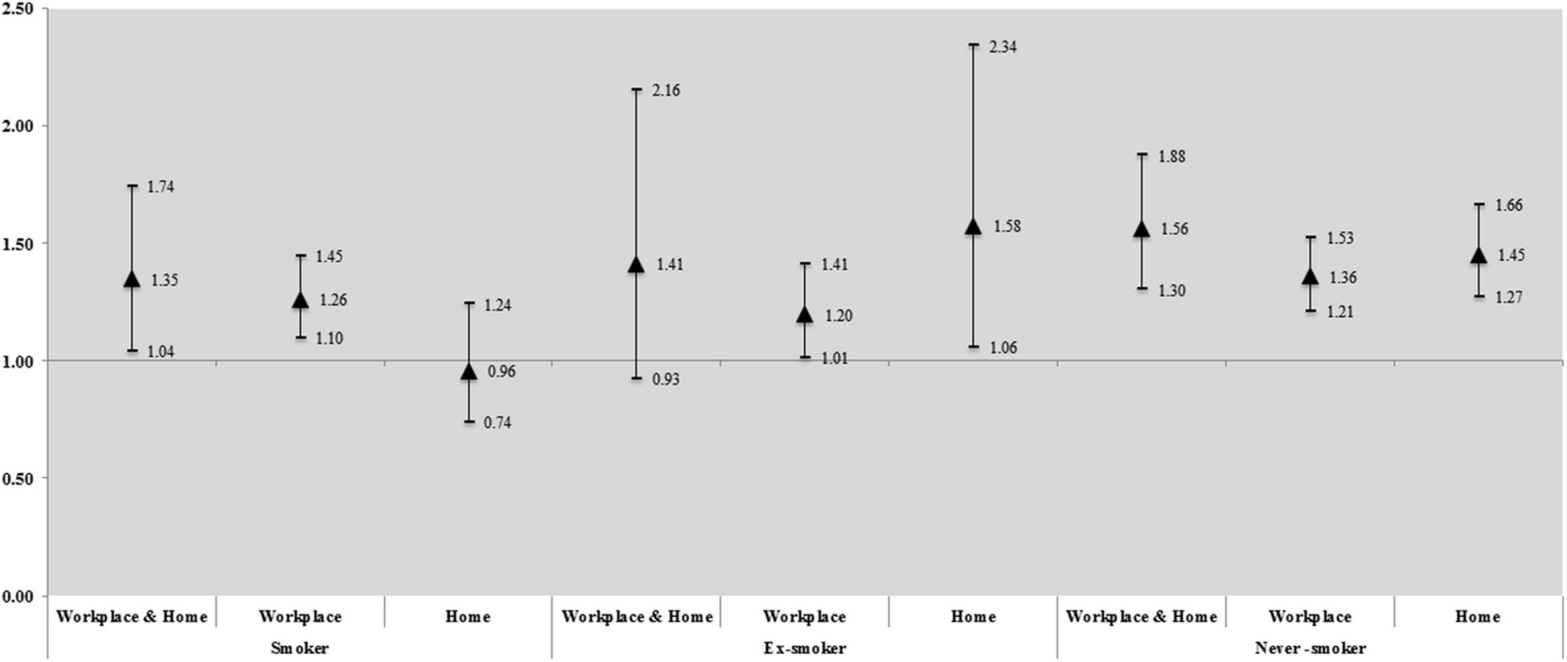
Results of logistic regression, indicating the association between exposure to SHS in different locations and stress according to smoking status. *All variables are adjusted for marital status, age, income, economic status education level, perceived health status, and year

The second outcome, the association between different exposure locations and stress according to smoking status, is shown in Fig. 2. In smokers, SHS exposure both in the workplace and at home (OR: 1.35; 95 % CI: 1.04–1.74) and in the workplace only (OR: 1.26; 95 % CI: 1.10–1.45) was more associated with higher stress than non-exposure. Among ex-smokers, the effect of SHS on stress was highest when exposure occurred at home (OR: 1.58; 95 % CI: 1.06–2.34). Never-smokers felt the least stressed when exposed to SHS, regardless of the location. However, the effect was highest when exposed to SHS both in the workplace and at home (OR: 1.56; 95 % CI: 1.06–2.34; Fig. 2).

## Discussion

The aim of our study was to assess the association between SHS exposure and stress. We found that almost half of the participants were exposed to SHS, with exposure in the workplace occurring more prevalently than at home. Although the intensity of the effect of SHS on stress varied according to smoking status, SHS was associated with an increase in stress. Particularly, the effect of SHS on stress was greatest in never-smokers. These results indicate that many people in Korea are exposed to unwanted SHS in their own homes and the workplace, suggesting that these locations are key sources of harm. Previous studies also suggested that the location of SHS is important (e.g., bars, restaurants, public places, homes) [29–32]. Such locations are closely connected with personal everyday life, meaning that the potential for exposure to SHS also increases when visiting such locations. It also implies that designating smoke-free areas in such locations is important for reducing SHS.

Thus, we performed a further evaluation that compared SHS exposure in the workplace and at home. Our results indicated that the effect of SHS on stress differed according to both location and smoking status. Among smokers, exposure to SHS in the workplace and at home was associated with an increase in stress. However, the effect of SHS on stress decreased when smokers were exposed only in the workplace. This increase in stress among smokers may have been associated with the effects of nicotine. Smokers undergo physical changes associated with smoking that are similar to those that occur when people experience stress [33–35], as nicotine leads to increased heart rate, blood pressure, and breathing rate [26]. Thus, the chronically high nicotine levels in the bodies of smokers compared to non-smokers may mean that smokers have a greater physical reaction to SHS exposure that makes them feel more stressed. However, ex-smokers were found to experience a greater effect of SHS on stress when they were exposed to SHS at home only; conversely, a lesser effect was found for SHS exposure in the workplace. Ex-smokers may find it difficult to quit smoking due to the nicotine addiction associated with smoking [36]. Therefore, an ex-smoker (who does not currently smoke) may feel stressed when exposed to SHS, as it may tempt him or her to smoke. A previous study suggests that exposure to SHS is an important factor in determining the success of people who attempt to quit the smoking [37, 38]. Similar research has suggested that, in Korea, it is difficult to quit smoking when living with a smoker [39]. Thus, ex-smokers who are exposed to SHS at home might be hindered in their intention to quit smoking, which could account for the increase in stress. Among never-smokers, exposure to SHS was associated with a high increase in stress, both in the workplace and at home. These results suggest that the restriction of smoking both in the workplace and at home may be important to reducing stress. This finding is similar to a previous study that reported that SHS was found to have a negative effect on mental health [14, 40]. However, certain studies contrasted with our study in that they were unable to find an association between mental health and exposure to SHS [41, 42].

In Korea, there has been a ban on smoking in buildings of a certain size, which has contributed to an increase in the number of smoke-free areas. However, this ban does not currently apply to all indoor locations and is weaker than those of other countries. As a result, there has been no decrease in SHS, even after the implementation of a smoke-free law [43, 44]. Although many public places and other indoor locations have been designated as smoke-free areas, many people in Korea continue to face problems related to SHS. These results suggest that stronger regulations related to smoking bans are needed in Korea. Restrictions calling for smoke-free areas should also include exact evaluations as to how to create separate smoking areas. In Korea, public places that have designated smoke-free areas must also create areas for smokers. However, such smoking areas were often found to not be completely separate from smoke-free areas, resulting in the occurrence of SHS in smoke-free areas [45]. Thus, policies allowing for smoking areas must ensure that such areas are completely separated from smoke-free areas in the future. To do so, an employer must establish strict smoking regulations in the workplace and provide a separate space for smokers. In addition, better regulations for ensuring smoke-free areas in homes are needed in order to reduce domestic SHS. In Korea, smoke-free areas have primarily been implemented in public places and locations used by many people, and regulations calling for smoke-free areas in private places are lacking in comparison. Such differences might be due to the social aspect of smoking, as, generally, few home smoking restrictions have been implemented in Korea [46]. Thus, a public campaign for improving awareness of the risk of SHS on health is needed in order to reduce unwanted SHS at home. In addition, children should be educated about the risks of both smoking and exposure to SHS.

There were a number of limitations in this study. First, our study was cross-sectional, and information was obtained via self-report, meaning that we were unable to imply causation between stress and exposure to SHS. Self-report can lead to an underestimation of the actual exposure to SHS and might be associated with recall bias [47]. However, a previous study suggested that self-report had high validity and that results were similar to those obtained from biological markers [48]. Second, our study did not investigate SHS exposure other than that in the home and workplace, and the exposure duration in each of these locations was not assessed. Therefore, future studies should investigate other public places of SHS exposure, such as at bus stops and on streets. Third, we did not consider the potential effect of the workplace environment on stress. Finally, unmeasured variables and other potential stressors, such as relationships with co-workers and spouses, could have confounded the present results. Thus, a further study considering such factors is needed.

However, this study had several strengths. We used KNHANES data, which ensured that a reliable sampling design was implemented countrywide. Additionally, the large scale of the survey meant that it was representative of the nation as a whole. Second, although many studies have focused on the physical health effects of SHS exposure, our study was the first to focus on stress, a negative mental effect, in relation to SHS exposure. Finally, we suggest that exposure to SHS may be an important risk factor for stress, particularly if no interventions to prevent SHS are undertaken.

In our study, we found that SHS exposure in a specific location may increase individual stress. Both the workplace and the home are important places, as they are closely connected to life and can have a major influence on each individual. Therefore, the enforcement of smoke-free policies and laws banning smoking is essential for reducing unnecessary stress, and policymakers should strengthen smoke-free regulations to reduce unwanted stress related to SHS exposure.

## Conclusions

In conclusion, SHS may be a preventable risk factor for stress that should be managed appropriately. Consistent management of smoke-free areas would result in a healthier environment. Thus, policymakers should consider anti-SHS policies in order to reduce preventable stress.

### Data source

The data used in our study are open data. Anyone who submits a suitable form can use the data via website (https://knhanes.cdc.go.kr/knhanes/index.do).
